# PHYSICAL ACTIVITY INTERVENTION IN HOME CARE: COMPARING FAMILY AND NON-FAMILY CAREGIVING DYADS

**DOI:** 10.1093/geroni/igz038.733

**Published:** 2019-11-08

**Authors:** Naoko Muramatsu, Lijuan Yin

**Affiliations:** University of Illinois at Chicago School of Public Health, Chicago, Illinois, United States

## Abstract

An increasing number of states pay family members who care for older adults in Medicaid-funded home care. Previous research documented pros and cons of hiring family members as home care providers. However, little is known about whether family and non-family caregiving dyads function differently when they participate in health promotion interventions in home care. Using data collected in a pilot study of a gentle physical activity program delivered by home care workers in a Medicare home care program, this study compared 18 family and 32 non-family caregiving dyads in client outcomes (self-reported and performance-based function) and process outcomes (exercise-related social support provided by home care aides) before and after the intervention. Linear mixed models indicated that client outcomes improved after the 4-month intervention (p<0.05), controlling for clients’ age, gender, and number of chronic conditions. Compared to family caregiving dyads, greater improvement in exercise-related support was observed in non-family caregiving dyads (p<0.05), where care providers offered less exercise-related support at baseline. The intervention program was received well by both family and non-family dyads, as expressed by one of the caregivers: “It makes my client feel good about herself. I also feel good for my client.” Results suggest that empowering caregivers with health promotion skills is a promising strategy, especially in non-family caregiving dyads. Further research is warranted to produce evidence-based health promotion programs for family and non-family caregiving dyads in home care.

